# How Coca-Cola Shaped the International Congress on Physical Activity and Public Health: An Analysis of Email Exchanges between 2012 and 2014

**DOI:** 10.3390/ijerph17238996

**Published:** 2020-12-03

**Authors:** Benjamin Wood, Gary Ruskin, Gary Sacks

**Affiliations:** 1 Global Obesity Centre, Deakin University, Burwood, VIC 3125, Australia; gary.sacks@deakin.edu.au; 2 U.S. Right to Know, Oakland, CA 94611-5221, USA; gary@usrtk.org

**Keywords:** conference sponsorship, food industry, The Coca-Cola Company, corporate political activity

## Abstract

There is currently limited direct evidence of how sponsorship of scientific conferences fits within the food industry’s strategy to shape public policy and opinion in its favour. This paper provides an analysis of emails between a vice-president of The Coca-Cola Company (Coke) and prominent public health figures in relation to the 2012 and 2014 International Congresses of Physical Activity and Public Health (ICPAPH). Contrary to Coke’s prepared public statements, the findings show that Coke deliberated with its sponsored researchers on topics to present at ICPAPH in an effort to shift blame for the rising incidence of obesity and diet-related diseases away from its products onto physical activity and individual choice. The emails also show how Coke used ICPAPH to promote its front groups and sponsored research networks and foster relationships with public health leaders in order to use their authority to deliver Coke’s message. The study questions whether current protocols about food industry sponsorship of scientific conferences are adequate to safeguard public health interests from corporate influence. A safer approach could be to apply the same provisions that are stipulated in the Framework Convention on Tobacco Control on eliminating all tobacco industry sponsorship to the food industry.

## 1. Introduction

The processed food and beverage industry (hereinafter the food industry) has been identified as a major contributor to the global burden of obesity and diet-related diseases such as type 2 diabetes [1,2]. A growing evidence base highlights how the food industry attempts to shape public policy and public opinion in their favour, and concern has been raised about how the political influence of powerful food and beverage corporations poses a substantial challenge to public health efforts to prevent diet-related disease and improve population diets [3,4].

One of the key political strategies of the food industry involves shaping the evidence and framing the debate on public health issues in ways that favour corporate interests [4]. This discursive strategy of the food industry is similar to that used by the tobacco industry [1,5]. Scholars examining the strategies of the tobacco industry have shown how, for decades, the tobacco industry denied the association between smoking and lung cancer and continue to use a number of tactics to shift policy debates away from the health implications of smoking [5,6].

The Coca-Cola Company (Coke) is the largest global manufacturer of sugar-sweetened beverages (SSBs). SSBs have been linked to increasing rates of diet-related disease at the population level [7,8,9,10,11,12,13,14,15]. Recent work has uncovered a number of tactics that Coke has employed to shape the evidence and frame the debate on diet- and nutrition-related issues. These tactics include the sponsorship of academic research and research networks (e.g., the International Study of Childhood Obesity, Lifestyle and the Environment (ISCOLE) and the Latin American Study of Nutrition and Health (ELANS)), the sponsorship of front groups (e.g., International Life Sciences Institute, Global Energy Balance Network, Exercise is Medicine), the fostering of close relationships with authoritative public health institutions (e.g., Coke’s cultivation of the Centers for Disease Control and Prevention), and influencing medical and science journalists [16,17,18,19,20,21,22,23,24,25,26]. To date, however, little research has examined how the food industry uses its sponsorship of public health conferences as a platform for their messaging strategies. This is despite the fact that the processed food industry sponsors and participates in many diet- and nutrition-related public health conferences [23,27,28]. In light of this gap, this study aimed to provide direct evidence of how Coke used its sponsorship of the 2012 and 2014 International Congresses of Physical Activity and Public Health (ICPAPH) as a platform to advance its commercial interests. This was achieved through the qualitative analysis of internal email communications between a senior executive of Coke and a number of prominent public health figures.

## 2. Materials and Methods

In 2015–2016, the nonprofit consumer and public health research group U.S. Right to Know (USRTK) filed four state public records requests (sometimes referred to as “freedom of information” requests) as part of a wide-ranging investigation into the food industry’s influence over matters related to public health. These requests were via the Colorado Open Records Act to the University of Colorado, via the Louisiana Public Records Act to Louisiana State University, via the West Virginia Freedom of Information Act to West Virginia University, and via the South Carolina Freedom of Information Act to the University of South Carolina. As part of these public records requests, USRTK sought communications between university faculty and Coke for the period 2012 to 2017. In response, USRTK received 36,931 pages of documents. The documents were manually screened by one of the authors (G.R.), in conjunction with a coworker, for evidence of food industry influence related to public health policy and practice, including food industry sponsorship of scientific conferences. During the manual screening process, seven documents that made reference to Coke’s sponsorship of ICPAPH were identified.

Only the emails that involved direct communication between Coke staff and public health researchers were included in the analysis (five out of the seven documents). Each of the five emails included Dr. Rhona Applebaum, then-Vice President and Chief Health and Science Officer of Coke. The public health researchers included in the emails, all of whom had received funding from Coke, were Steven Blair (a professor at the Departments of Exercise Science and Epidemiology & Biostatistics, University of South Carolina; cofounder of Global Energy Balance Network; then-Vice President of Exercise is Medicine), Kenneth Fox (a professor at the University of Bristol, Centre for Exercise, Nutrition, and Health Sciences), Peter Katzmarzyk (co-Principal Investigator (PI) of ISCOLE), Timothy Church (co-Principal Investigator of ISCOLE), James Hill (a professor at the University of Colorado; then-President of Global Energy Balance Network), and John Peters (a professor at the University of Colorado; then co-Vice President of Global Energy Balance Network). One email exchange also involved Pedro Hallal, an associate professor in Epidemiology, who was the chairman of the 2014 ICPAPH in Rio De Janeiro (Rio). The email exchanges occurred between 2012 and 2014 and were in reference to either the 2012 ICPAPH in Sydney or the 2014 ICPAPH in Rio. The email exchanges also referred to ISCOLE and Exercise is Medicine (EIM). ISCOLE is a Coke-sponsored study that is one of the largest multicountry studies examining factors contributing to childhood obesity [29,30]. EIM is a global partnership, in which Coke is a founding partner, that is managed by the American College of Sports Medicine. EIM promotes the idea that physical activity is integral in the prevention and management of the majority of medical issues [31].

We read all the included emails in chronological order. In a similar fashion to other research that has analysed internal food industry communications, we used thematic content analysis to code data [20,32]. Themes were selected using a deductive approach based on a well-established framework for examining corporate political activity (CPA) [4]. The CPA framework that we used describes six strategies used by the food industry in their efforts to influence public opinion and public policy to serve their commercial interest. These are information and messaging financial incentives, constituency building, legal strategies, policy substitution, and opposition fragmentation and destabilisation. Within each of these strategies, the food industry has been observed to use a number of different practices to further its corporate interests [4]. Aspects of the CPA framework that were relevant to this study are outlined in Table 1. Data from the emails were supplemented by documents detailing the scientific programs and accepted abstracts of the 2012 and 2014 ICPAPH. These were sourced using “Wayback Machine”, an initiative of the Internet Archive that allows users to view archived websites [33].

## 3. Results

Five email exchanges that were sent between February 2012 and June 2014 were analysed (see Table 2). The email exchanges highlight how Coke set out to influence the scientific agendas of the 2012 and 2014 ICPAPH. In addition, the email exchanges support the notion that Coke used the ICPAPH to promote the front groups and research networks it sponsored and to establish relationships with key public health leaders and organisations.

### 3.1. Shaping the Evidence Base and Framing the Debate on Diet- and Public-Health-Related Issues

In one of the emails from 2012, Applebaum shared an internal memo with Coke’s sponsored researchers containing messaging and talking points about Coke’s involvement in the 2012 ICPAPH. In this memo, Coke wanted to make it clear that:

“We [Coke] have no involvement in the deliberations about the agenda, topic areas and speakers […] When we host scientific symposia in conjunction with an event like ICPAPH, our role is to invite the experts and reimburse them for their travel and expenses only. We do not have a role in their choice of topics or the information they choose to present” (Email 2).

The email exchanges, however, challenge the veracity of Coke’s public relations statement. They show that Applebaum was involved in deliberations on the selection of topics to be presented by Coke’s sponsored researchers at both the 2012 and 2014 ICPAPH. Specifically, Applebaum pushed for the dissemination of research on topic areas that shift blame away from the role of the food industry and its products in contributing to diet-related disease. In an email relating to ideas for abstracts to be submitted for consideration in the 2012 ICPAPH program, Applebaum wrote to Professors Fox and Blair and encouraged them to consider submitting abstracts on behaviour change and energy balance, respectively. It can be seen that Applebaum’s recommendations were implemented. A review of the 2012 ICPAPH scientific program revealed that Fox presented a talk about using self-determination theory to promote physical activity, and Blair presented a talk during a Coke-sponsored session titled “We will never understand the obesity epidemic or how to deal with it, unless we better understand energy imbalance”.

Similarly, in an email exchange discussing topic ideas prior to the 2014 ICPAPH, Applebaum asked Professor Blair:

“Do you think another major source of bias in these studies [epidemiological studies of body weight and mortality] is that most of them completely ignore physical activity, or if they mention it, use very flawed and inaccurate PA [physical activity] data? Why not a session addressing this specific point(s)” (Email 3).

In the same email exchange that included researchers Church and Katzmarzyk, Applebaum wrote

“Obviously the key to all of this [referring to topic ideas for the 2014 ICPAPH] continues to be individual behaviour and motivation—Ideas?” (Email 3).

Applebaum also asked for potential topic ideas that could be based around the 2013 International Olympic Committee’s Consensus Statement that focused on physical activity and behaviour change:

“Lastly—based on the brilliance of the IOC Consensus Statement—any thoughts/ideas catalysing around the 5 strategies that we can do a deeper dive on—focusing on PA [physical activity] and NCD prevention” (Email 3).

The 2014 ICPAPH scientific program revealed that Professor Blair was coauthor of an accepted abstract that stated in its conclusion: “The results support our hypothesis that high energy expenditure [related to high levels of physical activity] rather than low energy intake [related to food consumption] is the gateway for weight maintenance and obesity prevention”.

### 3.2. Promotion of Coke-Sponsored Research Networks and Health Initiatives

The email exchanges demonstrate how Coke used the 2012 and 2014 ICPAPH to promote ISCOLE. Applebaum wrote to Professor Katzmarzyk, co-PI of ISCOLE, encouraging him to submit an abstract for both the 2012 and 2014 ICPAPH about ISCOLE. The conference agendas and abstract booklets show that Professor Katzmarzyk was selected to give a presentation on ISCOLE for the 2012 ICPAPH and was a coauthor of an abstract accepted for the 2014 ICPAPH that used ISCOLE data.

Coke also used ICPAPH as a platform to promote Exercise is Medicine (EIM). As its name suggests, EIM advocates the idea that physical inactivity is central to many types of illness, including obesity and type-2 diabetes—a message that aligns with Coke’s overall scientific agenda [34]. Regarding an email exchange about ideas for the 2014 ICPAPH, Applebaum wrote:

“Also thinking of a pre-conf [pre-conference workshop] on Ex [Exercise] is Medicine (or during the ICPAPH) if there’s the necessary time… With the global mix—it’s a great opportunity to drive awareness and hopefully support for more EIM in more countries. Currently up to 40 [countries]–only 167 to go” (Email 3).

### 3.3. Establish and Foster Relationships with Key Public Health Researchers and Leaders

The email exchanges suggest that Applebaum had close working relationships with Coke’s sponsored researchers. They also expose how Coke used their relationships with sponsored researchers (and the institutions that the researchers represented) to promote its scientific and public relations agenda at ICPAPH. For instance, the emails reveal how Michael Pratt, Senior Advisor for Global Health in the National Center for Chronic Disease Prevention and Health Promotion at the Center for Disease Control and Prevention (CDC), was prepared to deliver Coke’s message about EIM. Michael Pratt is a public health leader in the area of physical activity and health, having previously served as the Chief of the Physical Activity and Health Branch of the CDC and the leader of the CDC’s World Health Organization Collaborating Center for Physical Activity and Health. Additionally, the emails show that Applebaum had established a relationship with Pedro Hallal, then-Chairman of the ICPAPH in Rio. An email from Hallal to Katzmarzyk, with Applebaum in carbon copy, suggests that Hallal was supportive of Coke’s scientific agenda at the 2014 ICPAPH:

“Hi Peter (cc Rhona). Rhona [Applebaum] and I just had a phone call and decided to have a session on ISCOLE in ICPAPH as part of our sponsorship agreement. The session will last for 90 min and I do suggest you two think about 4–5 speakers (10 min each) for the session” (Email 4).

Applebaum indicated that Coke would provide funding to support participation in the conference, by responding to Hallal’s email:

“We will help with the travel… so full speed ahead” (Email 4).

At the 2014 ICPAPH, both Pratt and Hallal were speakers in a symposium about public–private partnerships during a symposium titled “Is the elephant still in the room? What next after the publication of the Lancet Physical Activity Series?”. The Lancet Physical Activity Series 2012 explored the impact of physical inactivity on the world’s major noncommunicable diseases and included contributions from Hallal, Pratt, Blair, and Katzmarzyk [35]. Notably, the use of the phrase “elephant in the room” in relation to public–private partnerships echoed Applebaum’s use of the same phrase in her related email to Coke’s sponsored researchers. This indicated how closely the framing of the content of the session at ICPAPH aligned to Applebaum’s vision of what it should look like.

### 3.4. Opposition Fragmentation and Destabilisation through Criticising Public Health Advocates

In response to Dr. Hérick de Sá’s Lancet article titled “Can Coca-Cola promote physical activity?”, it was seen in one of the private email communications that Appelbaum resorted to the use of derogatory language in reference to critics of Coke’s sponsorship practices:

“First—yes, we helped to sponsor the 5th Congress [the 2014 ICPAPH]. We have been a major sponsor since this Congress started 10 years ago… Second—We knew it was only a matter of time before a miscreant would write a comment re [regarding] our sponsorship… And yes, we will continue to sponsor key health and PA [physical activity] congresses in the future. [We] have a few lined up already—including the European Congress on Sports Science the first week in July [2014] in Amsterdam. We won’t let the bastards keep us down and the minority of agenda drivers must never win out over the majority of evidence-based researchers” (Email 5).

The use of such derogatory language (e.g., “miscreant” and “bastards”), even in private forums, may serve to discredit and sully the reputation of leading public health researchers and advocates who have publicly criticised Coke’s practices. This language is consistent with a strategy of fragmenting and destabilising groups, such as the public health community, that are likely to oppose particular food industry practices [4].

## 4. Discussion

In his Lancet article, Dr. de Sá wrote the following about what he observed at the 2014 ICPAPH in Rio:

“The sponsorship [of Coke] was not only financial; Coca-Cola was everywhere—at side meetings, in the sponsors’ hall, giving away its products and propaganda. At a time when sweetened soft drinks are recognised by independent organisations as a major cause of the present uncontrolled obesity pandemic, which notably affects children and developing countries, such as China, India, and Brazil, this is worrying” [36].

Consistent with both de Sá’s abovementioned observations and the relevant components of the CPA framework used to inform our analysis, this study provides direct evidence of Coke’s sponsorship of ICPAPH as a means of deploying its “information and messaging”, “constituency building” and “opposition fragmentation and destabilisation’ strategies”. First, as part of its “information and messaging” strategy, Coke was seen to select topics to be presented at the 2012 and 2014 ICPAPH, despite publicly stating the contrary. Thus, it can be argued that Coke effectively used the conference as a platform to frame the debate and shape the evidence base on diet- and public-health-related issues through the promotion of its sponsored research focusing on physical activity. Second, as part of its “constituency building” strategy, Coke was shown to use ICPAPH to promote its sponsored research networks and health initiatives, namely, ISCOLE and Exercise is Medicine, and to establish and reinforce relationships with prominent public health researchers and leaders. Third, Coke privately criticised a public health advocate using derogatory vernacular, consistent with the strategy of “opposition fragmentation and destabilisation”. These findings support and supplement previous work that has examined how the food industry attempts to shape public opinion and public policy to promote and protect its own private interests [4,16,17,18,19,20,21,22,23,24,25,26,32,37,38].

This study highlights concerns regarding industry sponsorship of public health, medical and other scientific conferences. First, it is difficult to prove or disprove the veracity of self-reported conflict-of-interest statements made by sponsors regarding their own role in setting conference agendas, given that such influence can be easily hidden. As this study has illustrated, there was a clear difference between Coke’s PR messaging and what was communicated in their internal email exchanges, and it was only through freedom of information requests that this discrepancy was uncovered. However, it can be argued that even in the case of honest disclosures, corporate influence can still exist in more covert forms that simple conflict-of-interest disclosures detailing conference sponsorship arrangements are unable to capture [21,39,40]. Coke, by virtue of being a grantor, exercises power over its grantees and can rely on less visible and more structurally embedded forms of power to influence its grantees to act in the company’s best interests [39,40,41]. This argument is supported by recent work that identified that the majority of food-industry-sponsored research presents conclusions favourable to their sponsor [16].

Another related concern is how the food industry uses the practice of sponsoring and disseminating research in scientific conferences to consolidate its discursive power—that is, the power to influence the political process through the shaping of norms and ideas [42,43]. Scientific conferences—generally perceived as trustworthy and legitimate sources of information—can confer political and scientific legitimacy on industry sponsors and their views, especially when corporate messaging can be organised to be delivered by authoritative figures [44,45]. Such legitimacy is an important source of, and can reinforce, corporate discursive power, thereby strengthening a company’s ability to shape public opinion and the policy process [46,47,48]. Specific to the findings of this paper, Coke is shown to use the authority of public health organisations such as the CDC and the respective public health leaders, including Michael Pratt, to deliver its messages at the ICPAPH. Therefore, from a discursive power perspective, Coke effectively used the conference as a platform to bolster its legitimacy in the realm of public health, as well as the legitimacy of its preferred framing of the policy debate on the issue of diet-related disease—i.e., promoting the importance of individual responsibility rather than government regulation and the need for solutions that focus on increasing physical activity rather than curbing unhealthy food consumption [45].

One potential solution to safeguarding scientific conferences from hidden or less visible forms of food industry influence, as well as to mitigate the potential adverse consequences of bolstered food industry legitimacy within the public health space, is to adopt an approach similar to that stipulated by the World Health Organization Framework Convention on Tobacco Control (FCTC). Article 1(g) of the FCTC calls for a comprehensive ban on tobacco sponsorship of “any form” to “any event, activity or individual” [49]. Some conference organisers, such as Public Health Association Australia (PHAA), have already adopted this approach as they do not accept any food industry sponsorship at all. In its sponsorship protocol, PHAA writes:

“Companies whose profits depend, at least in part, on manufacturing or production, distribution, advertising or promotion, marketing, sponsorship, retailing or representing products and practices that when used as intended can be problematic or harmful to public health are explicitly excluded as sponsors for PHAA activities” [50].

It is important to note, however, that limiting or eliminating food industry sponsors would make it more difficult for some conference organisers to obtain the funding necessary to conduct conferences. For instance, the conference organisers of the 2012 and 2014 ICPAPH would have needed to have found AUD100,000 (AUD was close to parity with USD for much of 2012) and USD249,467, respectively, from other sources if they chose instead to refuse Coke’s funding [51,52]. While it is beyond the scope of this paper to discuss the organising of scientific conferences in detail, we argue that solutions such as virtual conferences as a means of lowering costs and increased government funding of public health conferences and research could offset potential funding gaps incurred from reducing or eliminating food industry funding.

A key strength of this paper is that it analyses data that present direct evidence that Coke interfered in the scientific agendas and presentations of ICPAPH. This paper has two important limitations. First, this research is based on a limited number of pieces of communication. Second, the email exchanges occurred from 2012 to 2014 and may, therefore, not be indicative of current practice. In this regard, further research is recommended to examine the current state of food industry sponsorship of relevant academic and scientific conferences. That being said, one of the emails (Email 5) revealed that Coke was planning to sponsor events beyond the 2014 ICPAPH, including the 2014 European Congress on Sports Science. Furthermore, evidence from more recent public-health-related scientific conferences suggests that food industry sponsorship remains widely prevalent [53,54,55].

## 5. Conclusions

There is a clear conflict of interest between food and beverage companies that profit from the sale of unhealthy products and the public health aim of reducing the burden of obesity and diet-related diseases such as type 2 diabetes [1]. The public health, medical and scientific communities, including researchers in the field of physical activity and health, should be critical of food industry efforts to shape the evidence and frame the debate on diet- and nutrition-related diseases. This paper makes an important contribution to public health literature by providing direct evidence of the strategies used by food companies to exert their influence as part of their sponsorship of scientific conferences. Our findings have exposed that Coke deliberated with its sponsored researchers on topics to present at ICPAPH, despite publicly claiming otherwise, in an effort to shift blame for the rising incidence of obesity and diet-related diseases away from its products onto physical activity and individual choice. Furthermore, Coke used ICPAPH to promote its front groups and sponsored research networks and to foster relationships with public health leaders in order to use their authority to deliver Coke’s messaging. Given the findings, we argue that the dissemination of scientific knowledge through scientific conferences should be better protected from hidden and less visible forms of corporate influence. The model of eliminating tobacco industry sponsorship, as stipulated in the Framework Convention on Tobacco Control, could be applied to the food industry as well.

## Figures and Tables

**Table 1:** Relevant aspects of the Corporate Political Activities (CPA) framework [4] used to inform the thematic analysis.

Strategies	Practices	Mechanisms
Information and messaging	Frame the debate on diet- and public-health-related issues	Shift the blame away from the food industry, e.g., focus on individual responsibility, physical inactivity
	Shape the evidence base on diet- and health-related issues	Fund research, including through academics, own research institutions and front groups Pay scientists as advisers, consultants or spokespersons Participate in and host scientific events Provide industry-sponsored education materials Suppress or influence the dissemination of research Emphasise disagreement among scientists and focus on doubt in science Criticise evidence and emphasise its complexity
Constituency building	Establish relationships with key opinion leaders and health organisations	Promote public–private interactions, including philanthropic, transactional and transformational relationships Support professional organisations through funding and/or advertising in their publications Establish informal relationships with key opinion leaders
Opposition fragmentation and destabilisation	Criticise public health advocates	Criticise public health advocates personally and publicly
	Infiltrate, monitor and distract public health advocates, groups and organisations	Support the placement of industry-friendly personnel within health organisations

**Table 2:** Details of the email exchanges analysed in this paper.

	Start Date	Email Sender(s)	Relevant Email Circle Members	Topic
1	9 Feb 2012	Rhona Appelbaum, Peter Katzmarzyk (reply)	Rhona Appelbaum, Peter Katzmarzyk, Steven Blair, Kenneth Fox	Abstract submissions for ICPAPH 2012
2	8 Nov 2012	Rhona Appelbaum	Rhona Appelbaum, James Hill, John Peters	Coke’s PR messaging for ICPAPH 2012
3	20 Oct 2013	Rhona Appelbaum, Peter Katzmarzyk (reply)	Rhona Appelbaum, Peter Katzmarzyk, Timothy Church, Steven Blair	Research and session ideas for ICPAPH 2014
4	3 Nov 2013	Pedro Hallal, Rhona Appelbaum (reply), Peter Katzmarzyk (reply)	Pedro Hallal, Rhona Appelbaum, Peter Katzmarzyk	Planning of an ISCOLE session for ICPAPH 2014
5	13 June 2014	Rhona Appelbaum	Rhona Appelbaum, Peter Katzmarzyk, Timothy Church	Response to Dr. Hérick de Sá’s paper in the Lancet re: Coke’s sponsorship of ICPAPH 2014
